# Identification of Novel Genetic Loci Involved in Testis Traits of the Jiangxi Local Breed Based on GWAS Analyses

**DOI:** 10.3390/genes16060637

**Published:** 2025-05-27

**Authors:** Jing-E Ma, Ke Huang, Bahareldin Ali Abdalla Gibril, Xinwei Xiong, Yanping Wu, Zhangfeng Wang, Jiguo Xu

**Affiliations:** 1 Jiangxi Provincial Key Laboratory of Poultry Genetic Improvement, Nanchang Normal University, Nanchang 330032, China; majinge@ncnu.edu.cn (J.-E.M.); hk25755@126.com (K.H.); abdalla406@163.com (B.A.A.G.); xinweixiong@ncnu.edu.cn (X.X.); tmxk1965@163.com (Z.W.); 2 Institute of Animal Husbandry and Veterinary Medicine, Jiangxi Academy of Agricultural Sciences, Nanchang 330200, China; wyp_0902@126.com

**Keywords:** testis weight, candidate genes, GWAS, chicken, bioinformatic analyses

## Abstract

Background: The testis, a critical reproductive organ in male animals, is responsible for sperm production and androgen secretion. Testis weight often correlates with reproductive performance, yet the genetic factors influencing testicular traits in chickens remain unclear. Methods: Previous genome-wide association studies (GWAS) have identified key genes affecting testicular traits in Kangle Yellow chickens, along with the associated regulatory pathways and Gene Ontology (GO) terms, through bioinformatic analyses. In this study, we utilized the existing literature, full-length transcriptome data, and proteome analyses to select key candidate genes. Results: We identified 13 associated markers for chicken testicular traits with 262 candidate genes. Nine candidate genes were found to regulate chicken testicular traits referred to integrated analysis, including *CDH3*, *ZFPM1*, *CFAP52*, *ST6GAL1*, *IGF2BP2*, *SPG7*, *CDT1*, *NFAT5*, and *OPRK1*. Physical interactions among these genes were also observed, implicating mechanisms such as cell adhesion molecules and neuroactive ligand–receptor interaction. Conclusions: These findings provide a genetic basis for improving testicular traits in Chinese native chicken breeds.

## 1. Introduction

The testis is a vital reproductive organ in male animals, producing sperm and secreting androgen. Studies have demonstrated a significant correlation between testis weight and reproductive performance [1], where a larger testis size often predicts higher semen production [2]. Consequently, testis traits are valuable indices for fertility selection. Moreover, chicken testis has substantial medical and economic value as a by-product of the meat industry [3,4]. Thus, identifying genomic regions and mutations associated with chicken testicular weight (TW) could facilitate the selection of chickens with enhanced reproductive performance.

Over the past decade, genome-wide association studies (GWAS) have identified several genetic variants linked to testicular traits in various broiler lines (Supplementary File S1). For instance, 17 single nucleotide polymorphisms (SNPs) on chicken chromosome 1 (GGA1) and GGA7 were associated with TW in 12-week-old broilers [5]. Other studies have identified significant epistatic effects linked to TW in different chicken lines [6,7], highlighting several candidate genes across various chromosomes. TCF21 was significantly correlated with testis growth and development in male broilers [7]. In a different study, an F2 population was derived from Beijing-You chickens and a commercial broiler line. The authors identified one quantitative trait loci (QTL) located between 6.55 and 8.56 Mb on GGA13 related to TW and TWP. Especially the gene γ-aminobutyric acid A receptor, α 1 (GABRA1), was located near this region [2].

Kangle Yellow Chicken is a native poultry breed originating from Jiangxi province of China, renowned for its exceptional meat quality, which is well-adapted to challenging environments and stress resistance. Recognized as a protected local genetic resource, its production has gained economic significance in rural revitalization programs, bridging culinary tradition with modern consumer demand for authentic, traceable food sources. The breed’s growing popularity reflects a global trend toward appreciating slow-grown, ethically raised poultry with distinct regional characteristics. However, the previous results showed that the heritability for testicular weights is moderate to high [2]. The coefficient of variation for testicle traits at 22 weeks was 42.41–45.13% in Kangle Yellow chicken [8]. These findings suggested that molecular selection could improve the consistency of reproductive performance in these breeds.

This study aims to identify SNPs associated with TW and testis percentage of body weight (TWP) in the Kangle Yellow chickens. We specifically examine the genomic regions controlling testis traits, analyze candidate genes within these regions, and describe their potential functions. Understanding the genetic loci involved in reproduction could inform targeted breeding strategies, accelerating the development of healthy, high-quality local breeds.

## 2. Materials and Methods

### 2.1. Ethics Statement

All experiments involving animals were conducted according to the guidelines for the care and use of experimental animals established by the ethics committee of Nanchang Normal University (No. NCNU2021-006). The Laboratory Animal Management Committee of Nanchang Normal University also approved experimental animal work.

### 2.2. Experimental Birds and Phenotypic Data for GWAS

From June 2021 to November 2021, 102 Kangle Yellow chickens were collected in Jiangxi province. All the birds were provided by the Jiangxi Nanshi Science and Technology Co., Ltd. (Nanchang, China). According to the same nutritional requirements, chickens were raised under standard conditions of temperature, humidity, and ventilation, having free access to water throughout the whole period.

Blood samples and tissues, including testis and hypothalamus, were obtained from these roosters. The male birds were slaughtered at 22 weeks of age. DNA was purified using the Quick Gene DNA Whole Blood Kit (Qinke, Beijing, China). The obtained DNA was quality-controlled. The “Jingxin NO.1” 55K SNP microarray, which targets 52,180 SNPs, was used to analyze all individuals (Chinese Academy of Agricultural Sciences, China) [9].

All phenotypic data (Supplementary File S2) related to testis traits were measured at age 22 weeks. The body weight (BW), left testis weight (LTW), right testis weight (RTW), and total testis weight (TW) were recorded. Testis percentage (TP = TW/BW) of left testis (LTWP), right testis (RTWP), and total testis weight (TWP) were also measured.

### 2.3. RNA Extraction, Synthesis of cDNA and qPCR

To compare the relative mRNA expression levels of MAML2, OPRK1, ATP6V1H, and MRPL15 in hypothalamic tissue, as well as GAS8 and ERICH1 in testicular tissue, between the High testis weight group and the Light testis weight group, total RNA was extracted from frozen tissues of testis and hypothalamus samples using TransZol Up Total RNA Extraction Kit (TransGen Biotech, Beijing, China) according to the manufacture’s protocol.

cDNAs were synthesized using the EasyScript^®^ One-Step gDNA Removal and cDNA Synthesis SuperMix (TransGen Biotech, Beijing, China) following the manufacturer’s protocol. qPCR was carried out with the primer sets for MAML2, OPRK1, ATP6V1H, MRPL15, GAS8, and ERICH1, using β-actin as a control (Supplementary File S3). Gene expression was measured using PerfectStart^®^ Green qPCR SuperMix (TransGen Biotech, Beijing, China) and a Bio-Rad CFX96 instrument (Bio-Rad, Hercules, CA, USA) in triplicate. qRT-PCR reactions followed thermocycling conditions: 10 min at 95 °C followed by 40 cycles of 30 s at 95 °C, 30 s at 60 °C, and 30 s at 72 °C, and finally, at the melting temperatures. Relative gene expression was quantified using the 2^(−ΔΔ^^Ct^^)^ value (ΔCt = Ct of the target gene-Ct of β-actin; ΔΔCt = ΔCt of Light testis weight group − ΔCt of High testis weight group). All qPCR data were expressed as means ± SEM. Student’s *t*-test measured statistical differences.

### 2.4. Bioinformatic and Statistical Analysis

GWAS analysis was carried out in PLINK using the linear regression analysis method [10]. The threshold *p* value for declaring genome-wide significance was 5.0 × 10^−4^, considering the limited number of genome-wide significant SNPs in the previous study. The Manhattan plots of the *p* values for all SNPs associated with LTeW, RTeW, TeW, LTeP, RTeP, and TeP were plotted in PLINK using the linear regression analysis method [10]. Gene locations and information were mined from the GRCg6a (GCF_000002315.6).

A gene enrichment analysis was performed using KOBAS on 1 June 2024 (http://bioinfo.org/kobas/) [11]. A protein–protein interaction network was constructed using the STRING on 1 June 2024 (https://string-db.org/). The protein–protein interaction network was visualized using Cytoscape 3.7.1. To reveal the functional significance of identified genes, a literature search was conducted to retrieve studies published over the past 20 years from PubMed. Potential insights on the gene function were summarized through meta-analysis. The expression levels of the candidate genes were examined based on data from the full-length sequencing transcriptome (*PRJNA917678*), which came from a previous study using six 22-week-old Ningdu Yellow individuals with large or small testes. Four tissues were involved in the full-length sequencing transcriptome, including the hypothalamus, pituitary [12], liver, and testis. The protein expression of the candidate genes was also examined using testicular proteomic sequencing, which was also from the same individuals [13].

## 3. Results

### 3.1. Genome-Wide Association Studies of the Testicle Traits

**Figure 1:**
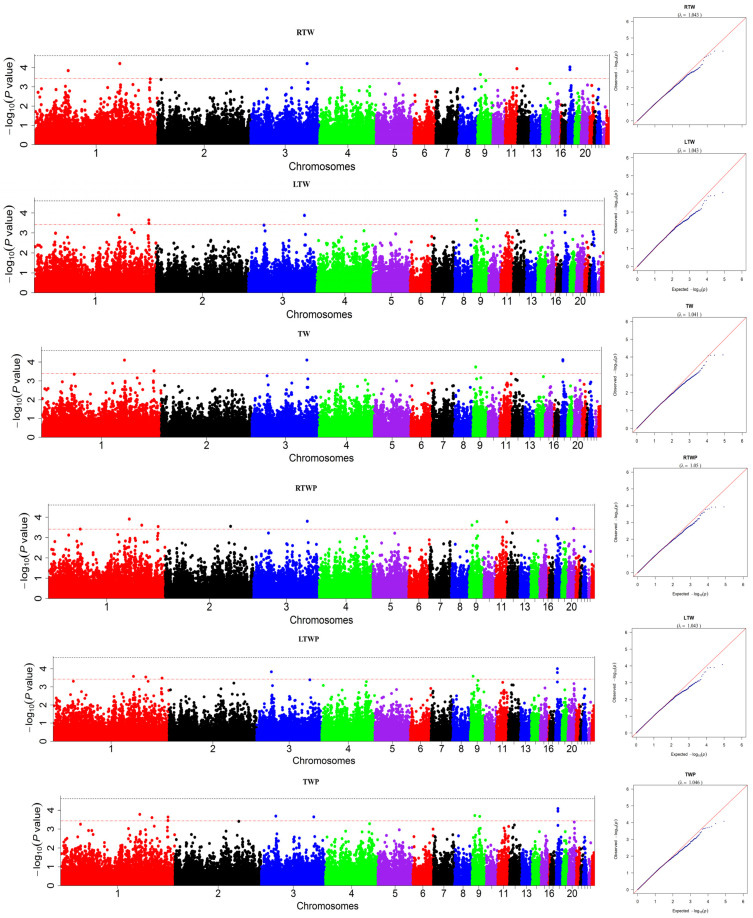
Manhattan and quantile–quantile (Q−Q) plots of GWAS results for six testicular traits in the Kangle Yellow chicken. The X−axis represents the chromosomes, and the Y−axis shows the corresponding −log_10_ *p* value. Abbreviations: RTW = right testis weight, LTW = left testis weight, TW= total testis weight, LTWP = testis percentage of left testis, RTWP = testis percentage of right testis, and TWP = testis percentage of total testis weight.

The association analysis was performed on a final filtered set of 40,560 SNPs. Thirteen SNPs were significantly associated with at least one of the six testicle traits (Figure 1). Five SNPs were located on chicken chromosome 1. Two SNPs were located on chromosome 3. There were also two SNPs on chromosome 9 and two SNPs on chromosome 18. One was on chromosome 11. The last one was on chromosome 2. Details of these SNPs are presented in Table 1.

There were seven SNPs associated with LTW, which were located on chromosome 1 (136,871,054, 185,445,943, 185,660,571), chromosome 3 (90,628,313), chromosome 9 (5,124,976), and chromosome 18 (3,169,740, 3,170,246). Seven SNPs were associated with RTW, which were located on chromosome 1 (53,862,399, 136,871,054), chromosome 3 (90,628,313), chromosome 9 (5,124,976), chromosome 11 (18,863,652), and chromosome 18 (3,169,740, 3,170,246). Six SNPs were associated with TW, which were located on chromosome 1 (136,871,054, 185,660,571), chromosome 3 (90,628,313), chromosome 9 (5,124,976), and chromosome 18 (3,169,740, 3,170,246). Seven SNPs were associated with LTWP, which were located on chromosome 1 (136,871,054, 157,924,199, 185,660,571), chromosome 3 (25,006,145), chromosome 9 (5,124,976), and chromosome 18 (3,169,740, 3,170,246). Ten SNPs were associated with RTWP, which were located on chromosome 1 (136,871,054, 157,924,199, 185,660,571), chromosome 2 (110,473,923), chromosome 3 (90,628,313), chromosome 9 (5,124,976, 13,678,170), chromosome 11 (18,863,652) and chromosome 18 (3,169,740, 3,170,246). Nine SNPs were associated with TWP, which were located on chromosome 1 (136,871,054, 157,924,199, 185,660,571), chromosome 3 (25,006,145, 90,628,313), chromosome 9 (13,678,170, 5,124,976), and chromosome 18 (3,169,740, 3,170,246). As shown in Table 1, ten SNPs were significant for more than one trait.

### 3.2. Identification of Candidate Genes

Near the 13 SNPs (about a 2 Mb region) on chromosomes 1, 2, 3, 9, 11, and 18, 262 annotated genes were present in the NCBI database (Supplementary File S4). The 196 named genes are given in Table 1. Chr9 13,678,170 was contained among the intron of the *FGF*12 gene, which was fibroblast growth factor 12 isoform X1. Chr18 3,169,740 and chr18 3,170,246 were located in the intronic region of B3GNTL1, which was predicted as UDP-GlcNAc, β Gal β-1,3-N-acetylglucosaminyltransferase-like protein 1 isoform X1. Regions on chromosome 1 were 52,862,399~54,862,399, 135,871,054~137,871,054, 156,924,199~158,924,199 and 184,445,943~186,660,571 based on the location of the five SNPs. The region on chromosome 2 was 109,473,923-111,473,923. Regions on chromosome 3 were 24,006,145~26,006,145 and 89,628,313~91,628,313; on chromosome 9 were 4,124,976~6,124,976 and 12,678,170~14,678,170; on chromosome 18 was 2,169,740~4,170,246. The region on chromosome 11 was 17,863,652~19,863,652 (Supplementary File S4).

### 3.3. Go and KEGG Analysis

A total of 65 enriched KEGG pathways and 747 GO terms were found to be related to these candidate genes (Supplementary File S5). It showed the top five terms of GO and KEGG pathways, including the nucleus, RNA polymerase II cis-regulatory region sequence-specific DNA binding, sensory perception of pain, positive regulation of transcription by RNA polymerase II, cytoplasm, ribosome, cell adhesion molecules (CAMs), retinol metabolism, phenylalanine, tyrosine, and tryptophan biosynthesis, and neuroactive ligand–receptor interaction in Table S2 (Supplementary File S6).

### 3.4. Gene Function Prediction

**Figure 2:**
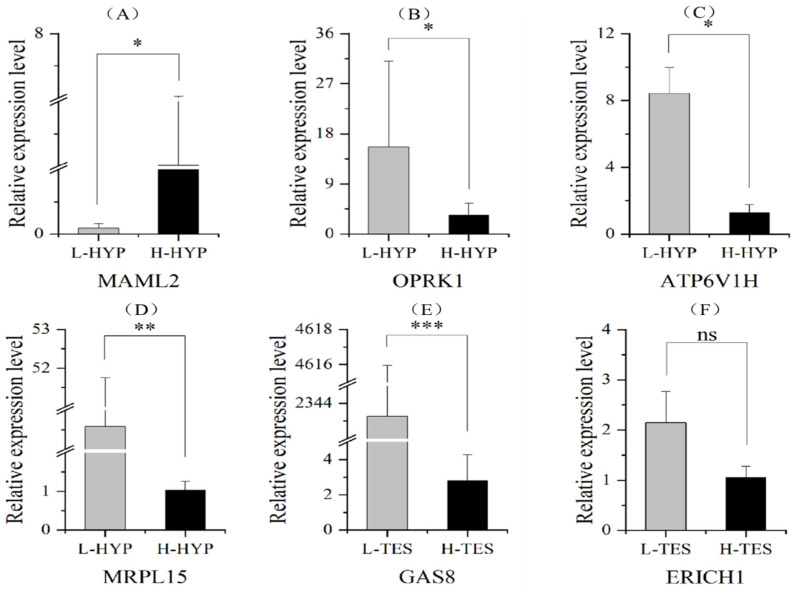
mRNA expression analysis of the six candidate genes identified by GWAS in Kangle Yellow chickens. (**A**–**D**) *MAML2*, *OPRK1*, *ATP6V1H*, and *MRPL15* are differently expressed in hypothalamus tissue between the High and Light testis groups. (**E**,**F**) *GAS8* and *ERICH1* are expressed differently in testis tissue between the High and Light testis groups.* means *p* < 0.05, ** means *p* < 0.01, *** means *p* < 0.001, ns means *p* > 0.05.

Based on full-length transcriptomic sequencing, 200 candidate genes were expressed in four tissues of six individuals with large or small testis, including the hypothalamus, pituitary, liver, and testis. A total of 38 genes were expressed differently in two groups with large or small testes, including 8 in the hypothalamus, 1 in the pituitary, 1 in the liver, and 30 in the testis (Table 2 and Supplementary File S7). A total of 6 genes out of the 38 genes were randomly selected, of which the expression level was tested to be differently expressed in the hypothalamus or testis in the two groups (Supplementary File S3 and Figure 2). Based on testicle protein sequencing, four proteins, including ONT.30993.1, ONT.31000.9, ONT.36147.5, and ONT.36153.15, were down-regulated in L-TES, corresponding to *CDK10*, *GAS8*, *CFAP52*, *TEKT3*, respectively.

Based on database mining and literature searches, 41 candidate genes out of 196 named genes were identified with known functions related to reproductive traits. These genes are shown in Table 3.

### 3.5. Protein–Protein Interaction Network

**Figure 3:**
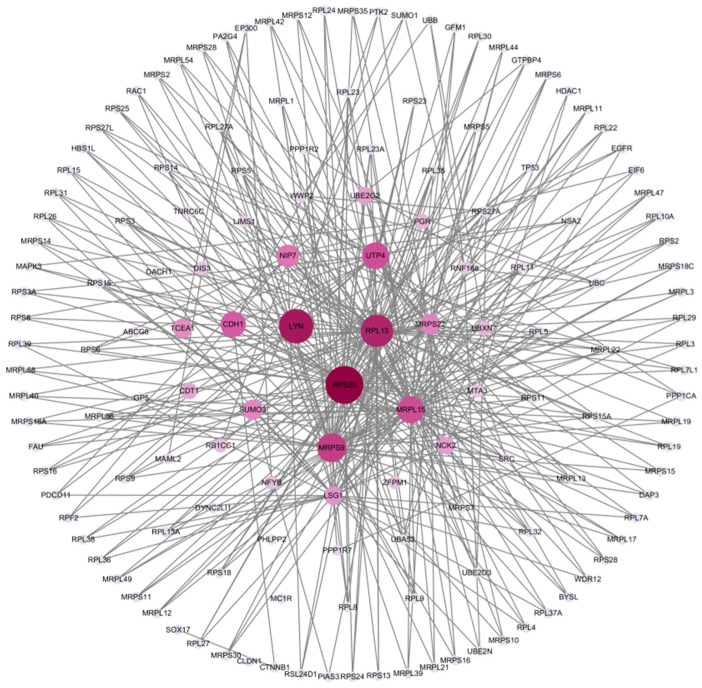
Result of gene interaction analysis of identified genes. The deeper color and bigger size of the plot represent the higher degree of the genes, and the thicker edge shows the closer of two genes.

An interaction network of the 262 candidate genes revealed 1347 links, with several genes, such as *RPS20*, *LYN*, *MRPS22*, *TCEA1*, *SUMO3*, *UTP4*, *RPL13*, *CDT1*, and *PPP1R2*, acting as central nodes (Figure 3).

## 4. Discussion

This study is the first to analyze six testicle traits at 22 weeks of age in the Jiangxi local chicken. A total of 13 SNPs were found to be significantly related to these traits located on Chr 1, 2, 3, 9, 11, and 18. A total of 262 candidate genes were detected by a GWAS. Our finding advanced the understanding of the genetic locus of testicle traits. Based on these genes, (1) GO and KEGG enrichment analysis was performed, and five significant GO terms and five KEGG pathways were selected. (2) The functions of 42 candidate genes were related to testicle traits referring to the literature. (3) A total of 38 genes and 4 proteins were also defied as potential candidate genes, the expression of which was significantly different in the tissues related to the HPT axis among the individuals with large or small testis based on the transcriptomic and proteomic sequencing data. (4) The network of protein interactions between the candidate genes found that 24 genes functioned as the nodes.

### 4.1. Genomic Region Analysis for Testicle Traits

The 13 SNPs were found on chromosomes 1, 2, 3, 9, 11 and 18. In previous studies, SNPs were also found in other regions of chr.1 or chr.3 that are significantly associated with testicular traits [5,7]. However, those SNPs were not similar to that of the previous studies. This might be related to the differences in the experimental material or testicular traits of different stages. However, our findings could complement the genetic basis of testicular traits related to mature roosters. Based on the current research, several gene loci related to testicular trait have been found on chromosomes 1, 3, 7, 9, 10, 11, 13, 18, 19, 21, and Z. Further study should be carried out on how these loci and the genes associated them interact each other to regulate testicular traits. According to our analysis, the association should be closely related between the testicular traits of local breeds and the SNP on chromosome 11. Genotype data analysis of the SNP is expected to be carried out among a larger group of roosters related to testicle traits.

### 4.2. The Gene Associated with Testicle Traits Related to GWAS

Nine key candidate genes were identified based on functional analysis in this study. These genes have been found to regulate testicular traits referred to in the literature. Eight genes were also found to be expressed differently in four tissues of two groups with large or small testes based on full-length transcriptome sequencing, including *CDH3*, *CFAP52*, *CDT1*, *IGF2BP2*, *ZFPM1*, *ST6GAL1*, *SPG7*, *NFAT5*, *OPRK1*, which were proved to be involved in the secretion of reproductive hormones, or the proliferation, differentiation and maturation of germ cells [19,25,28,35,40,46,47,49,61]. *CDH3* was reported to be expressed highly in the thyroid gland [64] and involved in the proliferation of mice germ cells [19]. *CFAP52* was reported to be expressed highly in the infundibulum and testis [64], affecting the blood barrier in the testis and sperm formation [23]. *IGF2BP2* was reported to be expressed highly in the ileum and pituitary, affecting sperm motility. *SPG7* participated in spermiogenesis [41], with a relatively high expression in the retina. *OPRK1* was highly expressed in the hypothalamus, playing a role in maintaining a normal GnRH pulse [61].

NFAT5 (nuclear factor of activated T cells 5) was also chosen as a candidate gene as a high-expression pituitary gene in chickens [54]. *NFAT5* was reported to act as a regulator of Wnt pathways, capable of binding to the promoter region of *WNT4*, modulating the reproductive process, and having the most vigorous activity and most significant response to FSH stimulation [54]. It was also found that *NFAT5* was up-regulated in the sperm of good osmo-adapters in Bos Taurus [53], which is related to the high fecundity in small-tail Han sheep [55]. *NFAT5* could be an epigenetic regulator of thermogenesis and obesity [56]. It could regulate not only the reproductive process but also animal weight gain. At this point, it was evident why this gene’s expression level was significantly correlated with testicle traits in this study. It revealed that *NFAT5* could affect testicular development through interaction with the reproductive hormone. The molecular mechanism could be explored in future studies.

*ZFPM1* is also highly expressed in the testicular tissue of chicken, which has been implicated in female fertility in a previous study [40]. *CDT1*, with a higher expression in the testis, was identified as a critical candidate gene controlling gonad development and germ cell maturation [65,66], leading to DNA replication and activating the cell cycle checkpoint during early embryo development [67]. These findings also provide evidence as to why its expression level could be correlated to the chicken body weight in this experiment. It showed that *CDT1* could regulate gonad development and other body parts as well. *ST6GAL1* was highly expressed in chicken testis. Recent studies have proved that it was associated with reproductive traits as regulators and biomarkers of sperm storage duration in egg layer breeders [46]. *ST6GAL1* was also identified as a biomarker for the implications of goat oligosaccharide biosynthesis [47]. The following studies should aim at its regulatory role in testicular traits.

### 4.3. Enriched Gene Pathway Analysis

In this study, the function of all the candidate genes was predicted through the GO and KEGG annotation. The most interesting result in our analysis was that several key candidate genes were also found in the enriched terms or pathways with the most significance. It contained RNA polymerase II cis-regulatory region sequence-specific DNA binding, sensory perception of pain, positive regulation of transcription by RNA polymerase II, cytoplasm, cell adhesion molecules, and neuroactive ligand–receptor interaction. Some of these genes are *ZFPM1*, *DACH1*, *NFAT5*, *CDT1*, *ZNF821*, *ZFHX3*, *SIX3*, *DZIP1L*, *CDH1*, *CLDN1*.

*DACH1* was known as a candidate gene for the reproductive traits of Chinese goats [68]. *ZFHX3* was described as a transcription factor that worked in various biological processes [69], which was essential for progesterone/progesterone receptor signaling [70], and the gene loci was one of the androgen receptor binding sites [71]. *Six3* has been implicated in regulating GnRH neurons linked to mouse infertility [72]. *CDH1* played a critical role in gonad oogenesis in the testis, promoting the migration and clustering of primordial germ cells with somatic cells [73]. *CLDN1* could regulate trophoblast apoptosis and proliferation in preeclampsia, a gestational hypertensive disease [74].

The findings suggest that the gene pathways on the candidate genes have significant implications for understanding the regulating pathways associated with testicle trait traits. Most of these genes were first reported to be important for chicken reproduction. The results also made it possible for us to see how these genomic regions could associate with the testicle traits.

However, the associated SNPs should be further verified in a larger population. The loci found in this study should also be tested to determine whether they are shared among the chickens. There was a limitation in that candidate genes near the SNPs still need further study, particularly the mechanisms by which these genes regulate testicular traits, which require further functional validation. Further exploration of these enriched entries could be meaningful for practicing molecular breeding in chickens by unlocking the underlying molecular mechanisms.

## 5. Conclusions

We identified 13 associated markers for chicken testicular traits with 262 candidate genes. Several genes were found to be important candidate genes based on utilized data from the existing literature, full-length transcriptome, and proteome analyses. Nine key candidate genes were identified to regulate testicular traits referred to previous reports in the literature, including *CDH3*, *ZFPM1*, *CFAP52*, *ST6GAL1*, *IGF2BP2*, *SPG7*, *CDT1*, *NFAT5*, and *OPRK1*. The interactions among these genes and their involvement in critical pathways, such as cell adhesion and neuroactive ligand–receptor interaction, suggest potential genomic strategies for improving testicular traits in Chinese native breeds. These SNPs and genes need further verification in the chicken population.

## Figures and Tables

**Table 1:** Significant SNPs and candidate genes associated with testicular traits in chickens identified through GWAS.

Chr	Position	Trait	Candidate Genes Related to Testicle Traits
1	136,871,054	TW, LTW, RTW, TWP, LTWP, RTWP	*ALDH1L2*, *APPL2*, *BPIFCB*, *C1H12ORF73*, *C1H2ORF40*, *C1H2ORF49*, *CCDC138*, *CCDC82*, *CKAP4*, *DACH1*, *DIS3*, *FAM76B*, *GCC2*, *GLT8D2*, *KLF12*, *LARGE1*, *LIMS1*, *MAML2*, *MIR1743*, *MIR6570*, *MIR7450*, *MRPS9*, *MTERF2*, *MTMR2*, *MZT1*, *NCK2*, *NFYB*, *NT5DC3*, *NUAK1*, *PGR*, *POU3F3*, *PRDM4*, *RTCB*, *SLC41A2*, *SLC5A7*, *SULT1C3*, *SYN3*
	185,660,571	TW, LTW, TWP, LTWP, RTWP	
	185,445,943	TW (L)	
	157,924,199	TWP, LTWP, RTWP	
	53,862,399	RTW	
2	110,473,923	RTWP	*ATP6V1H*, *CHCHD7*, *ERD2L*, *FAM150A*, *KIF20AL*, *LYN*, *LYPLA1*, *MOS*, *MRPL15*, *NPBWR1*, *OPRK1*, *PCMTD1*, *PENK*, *PLAG1*, *RB1CC1*, *RGS20*, *RP1RP1-2*, *RPS20*, *SDR16C5*, *SOX17*, *ST18*, *TCEA1*, *TGS1*, *TMEM68*, *TMEM68L*, *XKR4*
3	90,628,313	TW, LTW, RTW, TWP, RTWP	*ABCG8*, *CAMKMT*, *DYNC2LI1*, *MTA3*, *PLEKHH2*, *PPM1B*, *SIX3*, *SLC3A1*, *TRNAI-UAU*
	25,006,145	TWP, LTWP,	
9	5,124,976	TW, LTW, RTW, TWP, LTWP, RTWP	*A4GNT*, *ACAP2*, *AGXT*, *AMOTL2*, *APOD*, *ATP13A3*, *ATP13A4*, *ATP13A5*, *BDH1B*, *BOK*, *CEP19*, *CLDN1*, *COPS9*, *CPN2*, *DNAJB11*, *DTYMK*, *DZIP1L*, *FARP2*, *FGF12*, *GAL3ST4*, *GMNC*, *GP5*, *GPR35*, *GPR35L*, *GPR55*, *HES6*, *IGF2BP2*, *KLHL30*, *LRRC15*, *LSG1*, *MB21D2*, *MIR1577*, *MIR1608*, *MIR1612*, *MIR1704*, *MRPS22*, *OTOS*, *P3H2*, *PFKL*, *PPP1R2*, *PPP1R7*, *PTTG1IP*, *RNF168*, *RYK*, *SEPT2*, *SLCO2A1*, *SNED1*, *ST6GAL1*, *SUMO3*, *THAP4*, *TM4SF19*, *TMEM207*, *TRA2B*, *TSPEAR*, *UBE2G2*, *UBXN7*, *UTS2B*, *WDR53*, *XXYLT1*
	13,678,170	TWP, RTWP	
11	18,863,652	RTW, RTWP	*ACSF3*, *AP1G1*, *BANP*, *CDH1*, *CDH15*, *CDH3*, *CDK10*, *CDT1*, *CHST4*, *CPNE7*, *CTU2*, *CYB5B*, *DEF8*, *DPEP1*, *GAS8*, *HAS3*, *IL17C*, *JPH3*, *MC1R*, *MIR140*, *MIR1571*, *MIR6667*, *NFAT5*, *NIP7*, *PHLPP2*, *PMFBP1*, *RPL13*, *SNTB2*, *SPG7*, *SPIRE2*, *TAT*, *TCF25*, *TMCO7*, *TRAPPC2L*, *TUBB3*, *UTP4*, *VPS4A*, *WWP2*, *ZC3H18*, *ZFHX3*, *ZFPM1*, *ZNF276*, *ZNF469*, *ZNF821*
18	3,169,740, 3,170,246	TW, LTW, RTW, TWP, LTWP, RTWP	*B3GNTL1*, *C18H17orf62*, *CD7*, *CDRT1*, *CFAP52*, *FAM18B1*, *HS3ST3B1*, *OGFOD3*, *PMP22*, *RAB40B*, *SEPT9*, *TBC1D24L*, *TEKT3*, *TNRC6C*, *TRNAM-CAU*, *TRNAQ-CUG*, *TRNAQ-UUG*, *USP43*, *WDR45B*, *ZNF750*

**Table 2:** Thirty-eight candidate genes differentially expressed in four tissues of Kangle Yellow chickens with large or small testes were identified through full-length transcriptome sequencing.

Tissue	Count	Gene Name	
		Up-Regulated in L-TES	Down-Regulated in L-TES
Hypothalamus	8	*OPRK1*, *SYN3*, *PENK*, *FGF12*, *ATP6V1H*, *MRPL15*	*MAML2*, *PTTG1IP*
Pituitary	1	*WDR45B*	-
Liver	1	-	*CD7*
Testis	30	*CAMKMT*, *CDK10*, *CDRT1*, *DIS3*, *ERICH1*, *GAS8*, *KIF20AL*, *PMFBP1*, *PRDM4*, *SPG7*, *TEKT3*	*ACSF3*, *C18H17orf62*, *C1H12ORF73*, *CD7*, *CDH3*, *CDT1*, *DTYMK*, *FAM18B1*, *FARP2*, *GCC2*, *IGF2BP2*, *MRPL15*, *RNF168*, *ST6GAL1*, *TMEM68L*, *TRAPPC2L*, *USP43*, *XKR4*, *ZFPM1*

**Table 3:** Forty-two candidate genes associated with reproductive traits identified through database mining and literature searches.

Candidate Gene	Gene Description	Gene Function
*ALDH1L2*	Aldehyde dehydrogenase 1 family member L2	Functions in meiosis and regulating mouse fertility [14]
*ATP13A3*	ATPase 13A3	Related to enone levels in pigs [15]
*ATP13A4*	ATPase 13A4	Associated with bovine sperm motility [16]
*ATP13A5*	ATPase 13A5	Related to the freezing of bull sperm [17]
*CDH1*	Cadherin 1	A specific marker for undifferentiated spermatogonia in mouse testes [18,19]
*CDH3*	Cadherin 3	Related to the number of germ cells in mice [20]
*DNAJB11*	DnaJ heat shock protein family (Hsp40) member B11	Regulated the production of germ cells and pre-Sertoli cells of the developing gonad [21]
*MIR140*	MicroRNA 140	Affected testicular function through oxidative stress pathway [22]
*MIR7450*	MicroRNA 7450	Regulation of testicular development and spermatogenesis in geese [22,23,24]
*CFAP52*	Cilia and flagella associated protein 52	Affected blood-testis barrier and sperm formation [25]
*ABCG8*	ATP-binding cassette subfamily G member 8	Determined sperm flagellum morphology [26]
*AP1G1*	Adaptor-related protein complex 1 subunit γ 1	Affected spermatogenesis [27,28]
*CDT1*	Chromatin licensing and DNA replication factor 1	After deletion, mouse and zebrafish testicular epithelial cells were abnormal [29]
*CEP19*	Centrosomal protein 19	Involved in the ciliary assembly of the human sperm [30]
*CLDN1*	Claudin 1	Affected sperm morphology [31,32]
*COPS5*	COP9 signalosome subunit 5	Ensured the normal formation of the blood epididymal barrier in mammals [33]
*DZIP1L*	DAZ-interacting zinc finger protein 1 like	Related to sperm count and infertility [34]
*IGF2BP2*	Insulin-like growth factor 2 mRNA binding protein 2	Its mutation led to abnormal sperm flagellum morphology and affected sperm motility [35]
*PPP1R2* *PPP1R7*	Protein phosphatase 1 regulatory inhibitor subunit 2	In motile caudal sperm of mammalian, the association of PP1γ2 to PPP1R2 and PPP1R7 resembled immature caput sperm [36]
*SEPT2*	Septin 2	Associated with poor sperm motility in humans [37]
*SLC5A7*	Solute carrier family 5 member 7	Played an important role in mouse germ cell differentiation [38]
*ZNF750*	Zinc finger protein 750	Acted as a regulatory gene for the estrogen receptor in the rat [39]
*ZFPM1*	Zinc finger protein, FOG family member 1	Associated with regulation of testicular development and function in mice [40]
*VPS4A*	Vacuolar protein sorting 4 homolog A	Affected the progressive motility of spermatozoa in the Duroc boar population [41]
*UTP4*	UTP4 small subunit processome component	Affected fertility in Drosophila [42]
*UBE2G2*	Ubiquitin-conjugating enzyme E2 G2	Affected fertility in men [43]
*TUBB3*	Tubulin β 3 class III	Acted as a target of androgenic action on SCs from the initiation of meiosis to adult mice spermatogenesis [44]
*SUMO3*	Small ubiquitin-like modifier 3	Involvement of calcium in the regulation of centrin-1-SUMO-2/3 interaction in mouse testis [45]
*ST6GAL1*	ST6 β-galactoside α-2,6-sialyltransferase 1	As regulators and biomarkers of sperm storage duration in egg layer breeders [46,47]
*SPIRE2*	Spire-type actin nucleation factor 2	Associated with fertility traits in goats [48]
*SPG7*	SPG7 matrix AAA peptidase subunit, paraplegin	Participated in spermiogenesis by functioning in the mitochondria in Phascolosoma esculenta [49]
*SIX3*	SIX homeobox 3	Be required for female fertility [50]
*PMP22*	Peripheral myelin protein 22	As a negative regulator of spermatogenesis in mice [51]
*NFYB*	Nuclear transcription factor Y subunit β	Played a role in the self-renewal and proliferation of planarian SSCs [52]
*NFAT5*	Nuclear factor of activated T cells 5	Having the strongest activity and greatest response to FSH stimulation [53,54,55,56]
*NCK2*	NCK adaptor protein 2	Interacted with NCK2 to modulate human SSC self-renewal and apoptosis via cell cyclins and cycle progression [57]
*MTMR2*	Myotubularin-related protein 2	Affected the depletion of spermatids and spermatocytes from the seminiferous epithelium in man [58]
*MTA3*	Metastasis-associated 1 family member 3	Associated with steroidogenic dysfunction in mammals [59]
*LYPLA1*	Lysophospholipase 1	Regulating testosterone secretion [60]
*OPRK1*	Opioid receptor kappa 1	Playing a role in maintaining normal GnRH pulse [61]
*GAS8*	Growth arrest specific 8	Involved in sperm motility [62,63]

## Data Availability

The original contributions presented in this study are included in the article/Supplementary Material. Further inquiries can be directed to the corresponding author(s).

## Supplementary Materials

The following supporting information can be downloaded at https://www.mdpi.com/article/10.3390/genes16060637/s1, File S1: Several genetic variants linked to testicular traits in various broiler lines; File S2: The phenotypic data related to testis traits at age 22 weeks; File S3: Primer sequence information; File S4: 262 candidate genes near the 13 SNPs on chromosomes 1, 2, 3, 9, 11, and 18 present in the NCBI database; File S5: GO and KEGG annotation of the candidate genes; File S6: GO terms and KEGG pathways related to the candidate genes associated with testicular traits of Kangle Yellow chickens. File S7. The expression level of 38 genes in the 4 tissues, including 8 in the hypothalamus, 1 in the pituitary, 1 in the liver, and 30 in the testis, and 4 proteins in the testis of 2 groups with large or small testes.
