# Supplementary material for: Identification of Novel Genetic Loci Involved in Testis Traits of the Jiangxi Local Breed Based on GWAS Analyses
Source: Genes (Basel). 2025 May 27;16(6):637. doi: 10.3390/genes16060637 (PMC12193209; doi:10.3390/genes16060637)
Supplement: Supplementary file 1 [file genes-16-00637-s001.zip › Supplementary File S1.pdf]

Table S1 Several genetic variants linked to testicular traits in various broiler lines

Chr	SNP	Position (bp)	Reference genome	Candidate gene	Reference
1		96,567,610	GCF_000002315.6_GRCg6a	Intron region of GBE1	TW of F2 population constructed by crossing broiler cocks derived from Arbor Acres with high abdominal fat content and Baier layer dams (a Chinese native breed) at 12 weeks [5]
		96,603,222		Intron region of GBE1	
		97,110,674		Intergenic region between LOC101748186 and LOC101748284	
		97,346,214		Intron region of ROBO1	
		97,517,953		Intron region of ROBO1	
		97,769,053		Intron region of ROBO1	
		97,857,465		Intron region of ROBO1	
		97,872,500		Intron region of ROBO1	
3	Gga_rs14364352	59,757,561		MAP7, TCF21, EPB41L2	Testes traits of the fat and lean lines at 7 weeks [7]
	Gga_rs14364587	60,099,912		-	
	Gga_rs14705827	60,784,548		-	
	GGaluGA224607	61,317,809		-	
	Gga_rs14365191	61,522,182		-	
	GGaluGA224679	61,526,696		-	
	Gga_rs16286755	64,020,868		GJA1	
	Gga_rs16287113	64,522,837		-	
	GGaluGA225462	65,388,568		GPRC6A	
7		20,471,018	GCF_000002315.6_GRCg6a	Intergenic region between LOC101749123 and LOC107053823	TW of F2 population constructed by crossing broiler cocks derived from Arbor Acres with high abdominal fat content and Baier layer dams (a Chinese native breed) at 12 weeks [5]
		20,472,562			
		21,768,144		Downstream region of RBMS1	
		22,695,888		Intergenic region between LOC112532799 and LOC101749755	
		23,458,496		Exon region of LOC107053887	
		23,460,051		Exon region of LOC107053887	
		23,463,551		Exon region of LOC107053887	

		23,464,459	Exon region of LOC107053887	
		23,464,738	Exon region of LOC107053887	
	Gga_rs14748077	8,478,019	-	
	GGaluGA068209	8,928,839	TEX9	
	GGaluGA068594	10,330,735	-	
	GGaluGA068660	10,573,304	CYP19A1	
	GGaluGA068662	10,607,832	-	
	Gga_rs14703430	10,647,843	-	
	Gga_rs14005067	10,742,112	-	
	Gga_rs14946630	10,792,181	-	
	Gga_rs15575830	11,983,111	-	
	Gga_rs15575901	12,159,992	-	Testes traits of the fat and lean lines at 7 weeks [7]
10	Gga_rs14947056	12,323,517	-	
	Gga_rs14005930	12,487,480	PDE8A	
	Gga_rs14006710	13,195,918	SH3GL3	
	Gga_rs15569613	-		
	Gga_rs14003094	-		
	Gga_rs15574658	-		
	Gga_rs14002835	-		
	Gga_rs13678064	-		
	GGaluGA067711	-		
	GGaluGA067706	-		
	GGaluGA067693	-	RNF111, TCF12, ARPP19, MYO5A, MAPK6	Testes traits of the fat and lean lines at 7 weeks [6]
11	Gga_rs312924990<br>Gga_rs14958653	1.2-1.9 (Mbp)		Birds were from two boiler lines divergently selected for abdominal fat content [8]
13		6,550,000~ 8,560,000	NKX2-5D and GABRA1	Testes traits of Beijing-You chicken at day 93 [2]

19	Gga_rs15834332	-	C-C motif chemokine ligand 5 (CCL5), MIR142	Testes traits of the fat and lean lines at 7 weeks [6]
21	Gga_rs15179992	76,023	SDHB, PARK7, VAMP3	Testes traits of the fat and lean lines at 7 weeks [6]
	GGaluGA181809	87,719		
	Gga_rs15179999	113,769		
	Gga_rs10732124	125,790		
	Gga_rs15180005	140,660		
	Gga_rs15180007	145,617		
	GGaluGA181823	153,135		
	Gga_rs16176404	165,390		
	Gga_rs15180023	197,067		
	Gga_rs15180012	219,311		
	Gga_rs15180032	277,332		
	Gga_rs15180041	291,087		
	Gga_rs16176409	297,203		
	Gga_rs16176412	299,152		
	GGaluGA181852	304,979		
	Gga_rs16176425	320,390		
	GGaluGA181865	332,352		
	GGaluGA181868	332,687		
	GGaluGA181877	362,742		
	Gga_rs14281175	402,941		
	Gga_rs13602346	423,299		
	Gga_rs14281291	493,436		
	Gga_rs16176824	631,537		
	GGaluGA182048	647,587		
Z	Gga_rs14765324	37,246,321	AGTPBP1, CAMK4, CDC14B, FANCC, FBP1, GNAQ, PTCH1, ROR2 and STARD4	Testes traits of the fat and lean lines at 7 weeks (6)
	Gga_rs16768474	37,440,770		
	Gga_rs14765605	37,562,582		

Gga_rs16768723	38,066,346
Gga_rs14766107	38,424,448
GGaluGA351567	38,545,044
Gga_rs16047676	38,795,339
Gga_rs16781643	39,120,198
Gga_rs14745723	39,189,946
Gga_rs16131986	39,223,668
Gga_rs14787078	39,256,770
Gga_rs16781713	39,415,378
Gga_rs14787751	40,393,058
Gga_rs16782083	40,411,585
Gga_rs16754179	40,514,085
Gga_rs16132921	41,304,561
Gga_rs14015526	45,300,030
GGaluGA352176	45,695,927
Gga_rs14016510	46,745,968
