# Supplementary material for: Identification of Novel Genetic Loci Involved in Testis Traits of the Jiangxi Local Breed Based on GWAS Analyses
Source: Genes (Basel). 2025 May 27;16(6):637. doi: 10.3390/genes16060637 (PMC12193209; doi:10.3390/genes16060637)
Supplement: Supplementary file 1 [file genes-16-00637-s001.zip › Supplementary File S3.pdf]

Supplementary file S3 Primer sequence information

Primer name	Gene ID	Primer sequence	Product (bp)
β-actin-F	NM_205518.2	TCATTGTGCTAGGTGCCA	210
β-actin-R		CCTCTTCCAGCCATCTTT	
MAML2-F	XM_015280608.4	CCTTGCAACCAACAGAGTGC	159
MAML2-R		CCCAGTCCCAAAAGCAGCTA	
GAS8-F1	NM_001277617.2	ACAGCTGCAGGAGCACATTA	199
GAS8-R1		GATCTCCACTTGATGCCGCT	
OPRK1-F	NM_001318772.2	GCTGGTTGAAGCATTAGGCG	190
OPRK1-R		GGTGCTCTGCCTATCCATCC	
ERICH1-F	NM_001278029.2	ACAGTGTCTTGAGTCTCGCA	155
ERICH1-R		TGAACTTGGTATGATCAGGAGGC	
ATP6V1H-F	NM_001197326.2	CTATCCCCGTGGGAAACGAG	180
ATP6V1H-R		GTGGCGGTTTGAGGTTGTTC	
MRPL15-F	NM_001006388.2	AAAGCCGGAGAGAAGACGTG	171
MRPL15-R		CGGCAGCTATGTCCCTCATT	
