# Supplementary material for: Identification of Novel Genetic Loci Involved in Testis Traits of the Jiangxi Local Breed Based on GWAS Analyses
Source: Genes (Basel). 2025 May 27;16(6):637. doi: 10.3390/genes16060637 (PMC12193209; doi:10.3390/genes16060637)
Supplement: Supplementary file 1 [file genes-16-00637-s001.zip › Supplementary File S6.pdf]

Table S2. GO terms and KEGG pathways related to the candidate genes associated with testicular traits of Kangle Yellow chickens

Term	Count	*P*-value	Candidate genes associated with testicular traits
GO:0005634 Nucleus	40	1.9E-05	TRAPPC2L, WWP2, RYK, CDT1, SUMO3, ZFPM1, ZFHX3, NFYB, BOK, SIX3, ST18, KLF12, RGS20, PPM1B, CDK10, CPNE7, SOX17, DNAJB11, PGR, NUAK1, TCF25, TDRP, PLAG1, MTMR2, MTA3, APPL2, TGS1, VPS4A, FGF12, DACH1, LYN, KIF20AL, NFAT5, RTCB, PRDM4, DTYMK, CCDC82, ZNF750, HAS3, HES6
GO:0000978 RNA polymerase II cis-regulatory region sequence-specific DNA binding	11	6.2E-04	NFAT5, NFYB, ZFPM1, ZFHX3, SIX3, PRDM4, ZNF750, PGR, DACH1, PLAG1, ST18
GO:0019233 Sensory perception of pain	3	7E-04	OPRK1, MC1R, PENK
GO:0045944 Positive regulation of transcription by RNA polymerase II	16	7.2E-04	MC1R, WWP2, NCK2, NFAT5, ZFPM1, SOX17, PRDM4, KLF12, TCEA1, ZNF821, PGR, ZNF276, ZNF750, SIX3, PLAG1, ST18
GO:0005737 Cytoplasm	33	9.4E-04	TRAPPC2L, SNTB2, RYK, LYPLA1, SUMO3, ZFHX3, PHLPP2, LIMS1, WWP2, PPP1R7, RGS20, TEKT3, CPNE7, DNAJB11, NUAK1, CDH1, FGF12, CDH3, MTMR2, MTA3, SULT1C3, MC1R, PCMTD1, FARP2, TUBB3, DACH1, CAMKMT, THAP4, MOS, DZIP1L, PRDM4, DTYMK, HAS3
gga03010 Ribosome	4	1.8E-02	RPL13, RPS20, MRPS9, MRPL15
gga04514 Cell adhesion molecules	4	1.9E-02	CDH1, CLDN1, CDH3, CDH15
gga00830 Retinol metabolism	2	4.3E-02	ERD2L, SDR16C5
gga00400 Phenylalanine, tyrosine and tryptophan biosynthesis	1	4.7E-02	TAT
gga04080 Neuroactive ligand-receptor interaction	6	5.7E-02	MC1R, GPR35L, PENK, NPBWR1, OPRK1, GPR35
